# Investigation of the Link Between Food Assistance Programs and Physical Activity Among Children and Adolescents

**DOI:** 10.34172/jrhs.2024.162

**Published:** 2024-09-30

**Authors:** Pardis Noormohammadpour, Nicole Robertson

**Affiliations:** ^1^Dalla Lana School of Public Health, University of Toronto, Toronto, Ontario, Canada

**Keywords:** SNAP programs, Physical activity, Preschool children, Adolescents

## Abstract

**Background:** While the direct benefits of food assistance programs are well-documented, there is a need to explore indirect benefits like increased physical activity. This study examined whether participation in the Supplemental Nutrition Assistance Program (SNAP) was associated with improved physical activity levels in children and adolescents aged 2-17 in the United States during 2017-2018.

**Study Design:** A cross-sectional study.

**Methods:** This cross-sectional study used a subset of National Health and Nutrition Examination Survey (NHANES) data (n=2620). In the NHANES 2017-2018 dataset, physical activity was measured through self-report questionnaires, which captured participants’ frequency, duration, and intensity of various activities. We used weighted logistic regression and the Hosmer - Lemeshow - Sturdivant forward model - building strategy to investigate this hypothesized association using SAS version 9.4.

**Results:** In the adjusted model, controlling for the other variables in the model, we found that children and adolescents from households that had received SNAP/Food Stamps had 1.53 times higher odds (odds ratio [OR]=1.53, 95% CI: 1.24-1.89) of achieving the recommended guidelines of 60 minutes of daily physical activity compared to those who had not received benefits. Each additional year of age resulted in 0.82 times lower odds (OR=0.82; 95% CI: 0.79, 0.85) of meeting the recommended amounts of physical activity. Additionally, each unit increase in BMI was associated with 0.96 times lower odds (OR=0.96, 95% CI: 0.93, 0.98) of engaging in recommended physical activity.

**Conclusion:** These findings suggest that participation in the SNAP/Food Stamps program may indirectly benefit participants by increasing physical activity levels.

## Background

 Many studies have established the link between physical inactivity and poor health outcomes. Studies have indicated that physical inactivity is associated with many chronic conditions, such as diabetes, obesity, heart disease, and premature mortality.^1^ In contrast, physical activity has shown numerous benefits. Specifically in children and adolescents, physical activity has been shown to decrease the risk of the mentioned morbidities, improve cognition and performance in school, decrease stress, improve sleep, improve overall mental health, and ensure healthy growth and development, which can extend into adulthood.^1-3^ In order to maintain or improve overall health and reduce the risk of chronic disease, the Physical Activity Guidelines for American Children recommend a minimum of 60 minutes of aerobic, muscle-strengthening, or physical exercise daily.^4^ Despite this comprehensive knowledge of the benefits of physical activity, it is estimated that only one in four children in the United States (US) meet this recommendation, which has been further worsened by the COVID-19 pandemic.^1^ It is crucial to examine the factors contributing to physical inactivity in children and adolescents. Understanding these factors can help develop targeted interventions to overcome barriers to achieving recommended activity levels.

 Previous studies conducted in the US have suggested an association between lower socioeconomic status and physical inactivity in children^5-7^ and an inverse relationship between food insecurity and physical activity in children.^8,9^ Programs like Supplemental Nutrition Assistance Program (SNAP) might indirectly increase children’s physical activity by improving nutrition and freeing up household resources for activity-related expenses. Although these programs are not typically designed to boost physical activity, we aimed to explore this potential benefit. No prior research has examined the link between food benefit programs and physical activity levels in US children and adolescents. We focus on the SNAP program, the most significant anti- hunger initiative in the US, which supports over 41 million low-income individuals in accessing nutritious food.^10^

 The SNAP/Food Stamp program provides nutrition assistance to low-income individuals and families, offering a Benefits Card to purchase eligible food based on income and resource limits.^11^ Studies have suggested that the SNAP/Food Stamps Program has reduced food insecurity by as much as 30% while also putting money back into the economy, lessening the extent and severity of poverty, supporting low-paid workers, promoting healthy eating and improved health, and lowering health care expenditures.^10^ The program has been reported to have positive effects on the physical activity levels of children, which may be an important pathway to improve health outcomes.

 This study used a cross-sectional research design to explore whether participation in the SNAP/Food Stamps program was linked to improved physical activity in children and adolescents aged 2-17 years in the US during 2017-2018. Using publicly available data from the National Health and Nutrition Examination Survey (NHANES), the study aimed to quantify the relationship between SNAP/Food Stamp program participation and meeting recommended physical activity guidelines. The hypothesis was that exposure to the program would be associated with increased physical activity levels in the study population.

## Materials and Methods

###  Study population and data collection

 This cross-sectional analysis used a subset of the NHANES cohort, specifically all children and adolescents aged 2 to 17 years (2620 subjects) from the 2017-2018 cycle. The NHANES program, which has been designed to assess the health of children and adults across the US, combines interviews and physical examinations to examine a sample of approximately 5000 people per year across the country.^12^ The sampling methods aim to create a nationally representative sample of the US population, with oversampling of selected demographics as needed to produce reliable statistics^12^; the 2017-2018 NHANES cycle includes an oversampling of Asian Americans.^13^ We have chosen to study children and adolescents aged 2-17 because we believe that interventions targeting this priority group would greatly benefit population health now and into the future. The NHANES dataset used for this study can be accessed at https://wwwn.cdc.gov/nchs/nhanes/continuousnhanes/default.aspx?BeginYear=2017.

 We have used demographics, physical activity levels, food insecurity, and physical examination datasets retrieved from the National Centre for Health Statistics and merged them based on the participant ID. We excluded participants who had missing responses, refused to respond, or responded with “I don’t know” to the exposure of interest, namely the participation/receipt of the SNAP/Food Stamp program (ever received versus never received), and the outcome of interest, the number of days of being physically active for at least 60 minutes.

###  Measurements

####  Exposure 

 The exposure to be measured was the participation or receipt of SNAP/Food Stamp benefits. One adult in the participating household responded on behalf of all household members. This variable was assessed via the following question: “Have you or any member in your household ever received SNAP or Food Stamp benefits?”. Questions related to SNAP/Food Stamp benefits were collected at the household level.^14^ This will be treated as a binary variable with yes/no responses. Subjects who answered yes were considered to be exposed.^14^ Respondents who refused to answer, answered “I don’t know”, or did not provide a response to the question were considered to have missing data and were not included in the analysis.^15^

####  Outcome 

 The outcome to be measured was physical activity levels and the results were presented according to whether participants have met the physical activity guidelines of 60 minutes of daily physical activity.^16^ This variable was assessed via the following question, “During the past 7 days, on how many days were you physically active for a total of at least 60 minutes per day?”. Participants or their proxies (for children under the age of 12 or those who were unable to respond to the question themselves) were instructed to add up the time spent in any kind of physical activity that resulted in an increased heart rate and made the respondent breathe hard some of the time.^17,18^ Physical activity questions were asked by trained interviewers in the home using the computer-assisted personal interview system for participants aged 2 to 11 and 16 to 17 years; participants aged 12 to 15 were asked this question in the Mobile Examination Centre (MEC). Responses were then collapsed and dichotomized into a binary measure (yes or no) to decide whether they had met the guidelines; respondents were considered to have met the guideline if they reported being physically active for at least 60 minutes on seven days per week. Any lesser value was considered to have not met the guideline. Respondents who refused to answer, answered “I don’t know”, or did not provide a response to the question were considered to have missing data and were not included in the analysis.^15^

####  Covariates 

 Potential confounders were identified a priorithrough a literature review and conceptualized using a directed acyclic graph. Potential confounders were retrieved from the Food Security, Demographic Variables, and Body Measures NHANES datasets for the 2017-2018 cycle. Interview responses were collected through respondent-level and household-level interview data, and body measures were collected by trained health technicians in the MEC. We considered potential confounders related to the age of the participants and/or the household reference persons (numeric continuous variable), gender (categorical binary variable), height and weight or body mass index (BMI) (numeric continuous variables), race/ethnicity (categorical nominal variable), number of people in the household or in the family (numeric discrete variable), education level (categorical ordinal variable), marital status (categorical nominal variable), household income (categorical ordinal variable), and household food security category (categorical binary variable).

###  Statistical Analysis 

 All statistical analyses were conducted using SAS version 9.4 (SAS Institute Inc.). Given that the NHANES study includes a complex multistage probability sampling design to be representative of the civilian, non-institutionalized US population,^19^ examination sample weights provided by the NHANES database were used. The NHANES database recommends the use of the 2-year sample weights for all 2017-2018 analyses, and it recommends the use of the exam sample weights if household questionnaire data are merged with exam data.^13,17^ Further, as the primary sampling units and true design strata are not released to reduce the risks of disclosure and to protect the confidentiality of information provided by survey respondents,^13^ a masked variance unit (MVU) pseudo-primary sampling unit variable and an MVU pseudo-stratum variable were incorporated into the model for variance estimation.

 We ran descriptive statistics on our variables selected for possible inclusion based on our literature review and frequency, distributions, and missing data were assessed. NHANES recommends that if 10% or less of the data for the outcome variable are missing, it is usually acceptable to conduct the analyses without further adjustment^15^; therefore, we used 10% as our cut-off for missing data. We also reviewed the NHANES codebooks to confirm that skip patterns were not responsible for missing responses. Then, a Chi-squared test was used to compare participants with complete data on the exposure and outcome with participants who had missing data (those who responded “I don’t know”, refused to respond, or provided no response) for one or both of these variables.

###  Model selection

 We used the Hosmer-Lemeshow-Sturdivant (H-L-S) forward model building strategy^20^ to build our models. The H-L-S forward model-building strategy involves starting with a minimal set of predictors and systematically adding variables based on statistical criteria to improve model fit. This approach optimizes predictor selection and model performance while avoiding overfitting, ensuring a robust logistic regression model. As we were only interested in participants aged 2-17, we created a subset of the sample population to use for our analysis, as NHANES recommends subsetting data instead of removing observations that do not meet the inclusion criteria to provide correct variance estimates. Given that the outcome variable was initially coded as an integer value from 0 days to 7 days of physical activity of 60 minutes or more, crude and adjusted ordinal regression models were originally fitted. Variations of this ordinal model, including collapsing outcome categories and adding or removing covariates, were explored to check the appropriateness of this model and to determine if the proportional odds assumption was met. Then, multinomial regression and logistic regression models were used to compare the models to determine which would be the most appropriate. As there is a recommended guideline for physical activity in children and adolescents, it was determined that a binary logistic model would be used, assuming that the literature supports the dichotomizing of the outcome variable. Accordingly, crude and multivariable logistic regression models were used to determine the odds ratio and 95% confidence intervals for the crude and adjusted relationships between the SNAP and meeting the physical activity guidelines. An alpha level of 0.05 was used. All reported *P* values are one-sided.

## Results

###  Participant characteristics 

Table 1 presents the baseline characteristics of participants included in the subset of the study population. In total, 2620 participants were eligible for inclusion based on the age restrictions. In this age-restricted subset of the sample, the mean age of participants was 9.61 years. Additionally, 1304 (49.8%) of participants were male, and the mean BMI was 20.07 kg/m^2^. Of the 2527 participants or proxies that provided a response to the SNAP/Food Stamp Program question, 1451 (57.4%) of the participants had received SNAP/Food Stamp Benefits. Out of 2581 responses to the physical activity question, 1112 (43.1%) had met the physical activity guidelines. The majority of participants came from households that were considered to have full food security (n = 1404, 55.3%). Household reference persons were more likely to be married and/or live with a partner (n = 1854, 71.8%) and to have a high school diploma, a General Education Development (GED) certificate, a college degree, or an Associate’s degree (n = 1426, 57.3%). The median household size was five people (n = 662, 25.3%).

**Table 1:** Baseline characteristics of participants

**Quantitative variables**	**Mean**	**SE**	**Median, IQR**
Age of child (y)	9.61	0.14	9.14 (7.94)
Weight of child (kg)	42.03	0.54	37.13 (35.33)
Standing heightof child(cm)	138.59	0.65	141.19 (43.08)
Body mass index (kg/m^2^)	20.07	0.14	18.35 (6.45)
**Qualitative variables**	**Sample frequency**	**Weighted frequency**	**Weighted percent (%)**
Household food security benefit: ever received	2527		
Yes	1451	32 379 058	50.37
No	1076	31 901 452	49.63
Days of being physically active for at least 60 minutes	2581		
0	186	4 415 213	6.74
1	94	2 484 076	3.79
2	187	4 966 843	7.59
3	238	6 533 551	9.98
4	201	5 983 895	9.14
5	396	10 800 671	16.50
6	167	4 335 677	6.62
7	1112	25 939 777	39.63
Meet the physical activity guideline (60 minutes, 7 days per week)	2581		
No	1469	39 519 926	60.37
Yes	1112	25 939 777	39.63
Household food security category	2537		
Full food security: 0	1404	39 543 770	61.31
Marginal food security: 1-2	426	9 463 506	14.67
Low food security: 3-7	464	10 087 493	15.64
Very low food security: 8-18	243	5 407 194	8.38
Gender of child	2620		
Male	1304	33 682 101	50.82
Female	1316	32 590 322	49.18
Race of child	2620		
Mexican American	446	11 109 403	16.76
Other Hispanic	205	5 302 781	8.00
Non-Hispanic White	811	32 692 978	49.33
Non-Hispanic Black	620	9 006 956	13.59
Non-Hispanic Asian	277	3 269 246	4.93
Other races (including multi-racial)	261	4 891 059	7.38
Total number of people in the household	2620		
2	93	2 427 198	3.66
3	381	10 909 194	16.46
4	728	20 000 666	30.18
5	662	16 320 053	24.63
6	388	9 149 356	13.81
7 or more people in the household	368	7 465 957	11.27
Total number of people in the household	2620		
0-3	474	13 336 392	20.12
≥ 4	2146	52 936 032	79.88
Marital status of household reference person	2582		
Married/living with partner	1854	48 965 199	74.62
Widowed/divorced/separated	381	9 857 803	15.02
**Qualitative variables**	**Sample frequency**	**Weighted frequency**	**Weighted percent (%)**
Never married	347	6 797 182	10.36
Education level of household reference person	2490		
Less than high school degree	470	10 816 710	17.06
High school/GED/Associate’s degree	1426	33 750 332	53.24
College graduate or above	594	18 821 687	29.69
Household income ($)	2445		
0-4999	83	1 712 717	2.75
5000-9999	71	1 352 173	2.17
10 000- 14 999	100	2 271 593	3.64
15 000-19 999	149	3 203 459	5.13
20 000-24 999	144	2 893 754	4.64
25 000-34 999	290	6 062 935	9.72
35 000- 44 999	258	5 809 825	9.31
45 000-54 999	166	3 838 468	6.15
55 000-64 999	188	4 157 757	6.66
65 000-74 999	122	3 259 108	5.22
75 000-99 999	261	8 090 109	12.97
≥ 100 000	501	17 586 957	28.19
< 20 000	31	578 075	0.93
≥ 20 000	81	1 571 412	2.52

IQR: Interquartile range; SE: Standard error; GED: General Education Development.

###  Model fitting

 Upon testing the ordinal regression model and its variations, it was evident that the proportional odds assumption was violated (Brant test *P* < 0.001); therefore, this model was not an appropriate selection. Then, we explored the use of a multinomial logistic regression model to address the complex relationships in our dataset. However, the complex survey design of the NHANES dataset posed significant challenges. Specifically, in this design, the degrees of freedom for statistical tests are influenced by the number of primary sampling units rather than the total number of individuals sampled. Multinomial logistic regression is used when there is a categorical dependent variable with more than two levels. It requires a considerable amount of degrees of freedom to account for the multiple categories of the dependent variable and to adequately estimate the effects of the predictors on each category. In our case, the degrees of freedom needed for the multinomial logistic regression model exceeded the 15 degrees of freedom specified by NHANES^21^ for our dataset, which limited the robustness of our statistical analysis. The complexity of the NHANES survey design, including its clustering and stratification, meant that the effective sample size and degrees of freedom were reduced. The constraints imposed by these design features made it challenging to fit a multinomial logistic regression model appropriately, as the model required more degrees of freedom than were available. To address these constraints and ensure the robustness and reliability of our results, we opted for a binary logistic regression model. This model is more suited to situations where the dependent variable is binary and is less demanding in terms of degrees of freedom. By choosing the binary logistic regression model, we were able to adhere to the statistical limitations of the NHANES dataset while still providing meaningful insights into the relationships between the predictors and the outcome variable. The H-L-S method identified age, gender, BMI, and race/ethnicity of the participant as important covariates to be included in the model in addition to the exposure and the outcome. The H-L-S method also provided evidence that household food security level was not appropriate to be included as an effect measure modifier or as a confounding variable and therefore was not included. The Chi-square test demonstrated that the missing values for the exposure variable and the outcome variable were not related to one another (*P* = 0.41).

###  Associations between SNAP and physical activity

 In the crude logistic regression model, participants who received SNAP/Food Stamp benefits were 1.38 times more likely (odds ratio [OR] = 1.38, 95% confidence interval [CI]: 1.10, 1.72) to achieve the recommended physical activity guidelines than participants who did not receive SNAP/Food Stamp benefits. In the adjusted logistic regression model, controlling for the other variables in the model, participants who received SNAP/Food Stamp benefits were 1.53 times more likely (OR = 1.53, 95% CI: 1.24, 1.89) to achieve the recommended physical activity guidelines than participants who did not receive SNAP/Food Stamp benefits. Both findings were significant at the 0.05 level.

 Based on the adjusted model, controlling for the other variables in the model, male children were 1.35 times more likely (OR = 1.35, 95% CI: 1.11, 1.64) than female children to meet the recommended physical activity guidelines. Some demographic characteristics, such as age and BMI, were related to lower amounts of recommended physical activity. Mexican Americans had 0.58 times lower odds (OR = 0.58, 95% CI: 0.40, 0.83) of reaching the recommended amount of physical activity in comparison to non-Hispanic white subjects. Each year of increase in age resulted in 0.82 times lower odds (OR = 0.82, 95% CI: 0.79, 0.85) of meeting recommended amounts of physical activity. Moreover, the odds of engaging in recommended physical activity were 0.96 times lower (OR = 0.96, 95% CI: 0.93, 0.98) with each unit of increase in BMI. Results from the crude and adjusted models can be found in Table 2.

**Table 2:** Associations between selected variables and physical activity

**Variables**	**Unadjusted OR (95% CI)**	***P *** **value**	**Adjusted OR (95% CI)**	***P *** **value**
Age of child (y)	0.80 (0.78, 0.83)	0.001*	0.82 (0.79, 0.85)	0.001*
Body mass index (kg/m^2^)	0.87 (0.85, 0.88)	0.001*	0.96 (0.93, 0.98)	0.002*
Household food security benefit: ever received				
Yes	1.38 (1.10, 1.72)	0.008	1.53 (1.24, 1.89)	0.001*
No	Ref.		Ref.	
Gender of child				
Male	1.29 (1.09, 1.51)	0.005*	1.35 (1.11, 1.64)	0.006
Female	Ref.		Ref.	
Race of child				
Mexican American	0.65 (0.45, 0.92)	0.020*	0.58 (0.40, 0.83)	0.006
Non-Hispanic White	Ref.		Ref.	
Non-Hispanic Black	1.00 (0.71, 1.40)	0.990	1.00 (0.71, 1.40)	0.980
Non-Hispanic Asian	0.82 (0.49, 1.37)	0.420	0.83 (0.43, 1.59)	0.540
Other Hispanic	0.71 (0.45, 1.11)	0.120	0.56 (0.34, 0.92)	0.024*
Other races (including multi-racial)	0.89 (0.56, 1.42)	0.600	0.83 (0.54, 1.26)	0.350

CI: Confidence Interval; Ref: Reference Category *Statistically significant (*P* < 0.05) Odds ratios are adjusted for age, gender, BMI, race/ethnicity.

## Discussion

 These results provide evidence of the benefits of past or current participation in SNAP, or the Food Stamps Program, on child and adolescent physical activity levels. Those who received SNAP/Food Stamps benefits were found to have increased odds of meeting the physical activity guidelines of 60 minutes of daily physical activity, thus supporting our initial hypothesis. In addition, being a Mexican American or other Hispanic group, increasing age, or having a higher BMI were associated with lower odds of achieving recommended physical activity. On the other hand, the odds of reaching the recommended amount of physical activity were higher in males, controlling for the other variables in the model.

 These positive findings agree with the body of literature demonstrating the health benefits of the SNAP/Food Stamps program. In a recent study, Heflin et al showed that SNAP participation decreased all-cause mortality by about one to two percentage points throughout the population and decreased certain causes of death among persons aged 40 to 64.^22^ While the authors did not discuss the possible pathways behind their findings, our finding could explain a reason for their result if this association is also demonstrated in older age categories. Further, as participation in SNAP could increase the probability of meeting the recommended amount of physical activity in children and adolescents, the risk of chronic non-communicable diseases would decrease, which could lead to a decrease in the likelihood of all causes and specific causes of premature morbidity and mortality as adolescents transition into adulthood.

 Even though physical activity was assessed in different formats over various years in the NHANES databases, which provide a rich source of data regarding the amount of physical activity in the US population, the number of published studies in this field on children and adolescents remains relatively small. In 2014, To et al discussed that children who were food insecure did less moderate-to-vigorous physical activity than their counterparts who were not.^8^ In 2022, To et al demonstrated that physical activity, measured by a wearable device, was higher on weekdays than on weekends in children, and physical activity was affected by age, weight, and ethnicity.^23^ Further, previous studies have indicated that factors that influence physical activity may operate differentially across certain demographics, such as age, gender, and race/ethnicity.^24,25^ To the best of our knowledge, none of the previous studies investigated the effect of SNAP/Food Stamps benefits on meeting the recommended level of physical activity in children and adolescents. The findings of this study could be helpful to decision-makers regarding future policies on SNAP.

 To further substantiate our findings, we applied Hill’s considerations for causality.^26^ The significant odds ratios (OR = 1.53 for SNAP participation and physical activity) indicate a robust association, demonstrating the strength of this relationship. Our results are consistent with other studies showing the health benefits of SNAP participation,^22^ supporting the consistency criterion. The relationship between SNAP benefits and physical activity could be specific, with few other explanations, which may meet the specificity criterion. Additionally, SNAP participation may precede the increase in physical activity, which could suggest a temporal relationship requirement. There is some indications of a dose-response relationship, with higher SNAP benefits potentially correlating with increased physical activity, which may fulfill the biological gradient criterion. Improved nutrition and energy security from SNAP benefits provide a plausible mechanism for increased physical activity, addressing the plausibility criterion. The findings are coherent with the known benefits of SNAP in improving health outcomes,^27^ fulfilling the coherence criterion. While experimental data is limited, related interventions support our findings, addressing the experiment criterion. Finally, similar social support programs have shown causal relationships with health improvements,^28,29^ meeting the analogy criterion. By incorporating these considerations, we strengthen the argument that SNAP/Food Stamps benefits have a positive impact on physical activity levels in children and adolescents, independent of other covariates, as demonstrated by our adjusted analysis.

 One notable limitation of our study is the potential for underreporting related to the socioeconomic status of participants. Since SNAP is primarily used by individuals from lower socioeconomic backgrounds, there is a risk that some participants may be less inclined to provide accurate responses due to social stigma or discomfort with the topic. This could affect the reliability of the data and introduce bias into the study results. We acknowledge that this limitation may influence the validity of our findings and recommend that future research incorporate strategies to mitigate this issue, such as employing anonymized data collection methods. Additionally, 3.5% of the total participants had missing responses, refused to answer, or responded with “I don’t know” regarding SNAP participation. This limitation should be considered when interpreting the results. Another limitation to consider is that the average height and body mass index (BMI) of two-year-old children differ significantly from those of 17-year-old teenagers. Therefore, the results presented in Table 1 should be interpreted considering this limitation.

**Highlights**

- Children from SNAP households were 1.53 times more likely to meet exercise guidelines.
- Male kids were 1.35 times more likely than females to meet physical activity guidelines.
- Mexican Americans had 0.58 times lower odds of meeting physical activity guidelines.

## Conclusion

 In conclusion, the present study illustrated the higher odds of reaching the recommended level of physical activity in children and adolescents who have received SNAP. Additionally, meeting the recommended level of physical activity was associated with the age, gender, BMI, and race of participants. These results highlight the importance of the availability of supplemental nutrition programs for families that may be of lower income or experience food insecurity in the achievement of physical activity guidelines in children and adolescents and for improved health outcomes that extend into adulthood.

## Acknowledgments

 We would like to acknowledge Professor David Fisman for his valuable guidance.

## Authors’ Contribution

**Conceptualization:** Pardis Noormohammadpour, Nicole Robertson.

**Data curation:** Nicole Robertson, Pardis Noormohammadpour.

**Formal analysis:** Pardis Noormohammadpour, Nicole Robertson.

**Investigation:** Nicole Robertson, Pardis Noormohammadpour.

**Methodology:** Pardis Noormohammadpour, Nicole Robertson.

**Project administration:** Nicole Robertson, Pardis Noormohammadpour.

**Resources: **Pardis Noormohammadpour, Nicole Robertson.

**Software:** Pardis Noormohammadpour, Nicole Robertson.

**Supervision:** Pardis Noormohammadpour, Nicole Robertson.

**Validation:** Nicole Robertson, Pardis Noormohammadpour.

**Writing–original draft:** Nicole Robertson, Pardis Noormohammadpour.

**Writing–review & editing:** Pardis Noormohammadpour, Nicole Robertson.

## Competing Interests

 The authors have no conflicts of interests to declare.

## Ethical Approval

 As a publicly available dataset, NHANES does not require ethical approval for secondary analysis.

## Funding

 This study did not receive any funding or financial support.
